# Identification of hub genes for adult patients with sepsis via RNA sequencing

**DOI:** 10.1038/s41598-022-09175-z

**Published:** 2022-03-24

**Authors:** Qian Zhang, Yingchun Hu, Peiyao Wei, Liu Shi, Lei Shi, Jianzhou Li, Yalei Zhao, Yunru Chen, Xi Zhang, Feng Ye, Xiaojing Liu, Shumei Lin

**Affiliations:** 1 Department of Infectious Diseases, The First Affiliated Hospital of Xi’an Jiaotong University, Xi’an, Shaanxi China; 2 Department of Infectious Diseases, The Affiliated Hospital of Southwest Medical University, Luzhou, Sichuan China; 3 Department of Emergency Medicine, The Affiliated Hospital of Southwest Medical University, Luzhou, Sichuan China

**Keywords:** RNA, Genetics, Biomarkers, Bacterial infection

## Abstract

To screen out potential prognostic hub genes for adult patients with sepsis via RNA sequencing and construction of a microRNA–mRNA–PPI network and investigate the localization of these hub genes in peripheral blood monocytes. The peripheral blood of 33 subjects was subjected to microRNA and mRNA sequencing using high-throughput sequencing, and differentially expressed genes (DEGs) and differentially expressed microRNAs (DEMs) were identified by bioinformatics. Single-cell transcriptome sequencing (10 × Genomics) was further conducted. Among the samples from 23 adult septic patients and 10 healthy individuals, 20,391 genes and 1633 microRNAs were detected by RNA sequencing. In total, 1114 preliminary DEGs and 76 DEMs were obtained using DESeq2, and 454 DEGs were ultimately distinguished. A microRNA–mRNA–PPI network was constructed based on the DEGs and the top 20 DEMs, which included 10 upregulated and 10 downregulated microRNAs. Furthermore, the hub genes TLR5, FCGR1A, ELANE, GNLY, IL2RB and TGFBR3, which may be associated with the prognosis of sepsis, and their negatively correlated microRNAs, were analysed. The genes TLR5, FCGR1A and ELANE were mainly expressed in macrophages, and the genes GNLY, IL2RB and TGFBR3 were expressed specifically in T cells and natural killer cells. Parallel analysis of mRNAs and microRNAs in patients with sepsis was demonstrated to be feasible using RNA-seq. Potential hub genes and microRNAs that may be related to sepsis prognosis were identified, providing new prospects for sepsis treatment. However, further experiments are needed.

## Introduction

Sepsis is a complex syndrome involving host response malfunction and life-threatening organ damage caused by infection^1^. It is a common but severe emergency affecting approximately 19 million patients worldwide each year^2^. The incidence rate of sepsis in the ICUs of hospitals in China is approximately 20.6%, and the mortality rate of sepsis remains high despite the timely use of antibiotics and other beneficial adjutant therapies. The fatality rate of patients with severe sepsis can reach 50% or higher^3^. The pathogenesis of sepsis is complex and has long been the focus of medical research. The Multiple Organ Dysfunction (MOD) score and Sequential Organ Failure Assessment (SOFA) score are widely used for evaluation of organ damage and prognosis in patients with sepsis, but research has shown that do not adequately predict death or survival in individuals with sepsis^4^. Rapid diagnosis is an important means to improve the survival rate of sepsis and reduce sepsis-related organ dysfunction. At present, the diagnosis of sepsis mainly depends on recognition of clinical infection symptoms and blood cultures, for which there is a lack of rapid and sensitive biomarkers^5^. Currently, studies on biomarkers of sepsis are increasing. The level of procalcitonin (PCT) in patients with sepsis or septic shock at admission is considered to be a better prognostic indicator than other inflammatory markers^2^. Nevertheless, the critical value of PCT for determining death or survival in patients with sepsis cannot be determined^6^, and PCT cannot be used for sepsis prognosis prediction. C-reactive protein and white blood cells also exhibit poor specificity and sensitivity for the diagnosis of bacterial infection^7^. In particular, under the standard of Sepsis 3.0 standards, further study of the pathogenesis of sepsis and discovery of new biomarkers related to sepsis are urgently needed to provide a theoretical basis for clinical diagnosis and treatment.

High-throughput sequencing methods such as RNA sequencing (RNA-seq) have gained increasing attention with advances in science and technology and are now widely used in the transcriptomics field for applications such as including gene expression profiling, novel transcript discovery, and sequence variation detection^8^. RNA-seq can be applied to investigate different species of RNA, such as messenger RNA (mRNA), microRNA (miRNA) and long noncoding RNA, and it provides detailed insights into gene expression and the transcriptome. Combined with bioinformatic data analysis, RNA-seq is a promising approach for investigating different physiological and pathological conditions^9^. Compared with microarrays, RNA-seq has also shown great advantages; for example, it enables more accurate quantitative gene expression, requires fewer RNA samples, and enables the detection of transcriptome dynamics across different conditions^10^. In addition, RNA-seq has helped improve clinical diagnosis of patients with diverse diseases^11,12^. Using RNA-seq, it is feasible to investigate the key genes, miRNAs and immunometabolic features in sepsis^13,14^.

miRNAs are endogenous RNAs of approximately 23 nucleotides (nt) that play important roles in cell differentiation, growth, metabolism, cellular homeostasis, and other processes^15^. miRNAs regulate gene expression by binding to the 3′-untranslated regions (UTRs) or 5′-UTRs of their mRNA targets and alter transcriptional processes, as has been demonstrated in many diseases, including sepsis^16^. miRNAs are crucial regulators in the diagnosis and staging of sepsis and play a key role in determining the outcome of sepsis^17^.

RNA-seq is the gold standard for screening of differentially expressed genes (DEGs). However, selecting the core targets from thousands of DEGs is a challenge, so network analysis methods based on bioinformatics have become essential. Protein–protein interaction (PPI) networks can be applied to analyse the potential target genes in an integrated manner according to the principle of protein interactions, and a miRNA regulatory network can then be constructed based on miRNA-mediated posttranscriptional regulation of target genes. The joint construction of the two networks (which produces a miRNA–mRNA–PPI network) is expected to enable more efficient and accurate identification of potential core targets from multiple perspectives.

In the current study, we used RNA-seq to sequence miRNAs and mRNAs in peripheral blood cells of adult patients with sepsis in order to construct a sepsis miRNA–mRNA–PPI regulatory network. We screened the genes Toll-like receptor 5 (TLR5); FCGR1A; elastase, neutrophil expressed (ELANE); granulysin (GNLY); interleukin-2 receptor (IL-2R) β chain (IL2RB) and transforming growth factor beta receptor III (TGFBR3) as being associated with the prognosis of sepsis and identified miRNAs that negatively regulate these key genes. Furthermore, we confirmed the locations of the genes in peripheral blood mononuclear cells (PBMCs).

## Methods

### Subject recruitment and blood sample collection

Septic patients (n = 23) hospitalized in the EICU of the Department of Emergency Medicine at the Affiliated Hospital of Southwest Medical University from January 2019 to December 2019 were recruited for this study. Peripheral blood samples were collected from the septic patients and healthy volunteers (n = 10) using PAXgene Blood RNA tubes (BD Bioscience, San Diego, CA, USA) according to the manufacturer’s instructions and stored at − 80 °C in the Biological Sample Bank of the Affiliated Hospital of Southwest Medical University. The inclusion criteria were as follows: (1) diagnosis with sepsis and admission to the EICU; (2) compliance with the Sepsis 3.0 diagnostic criteria for sepsis (infection + SOFA score ≥ 2) published by the Society of Critical Care Medicine (SCCM) and the European Society of Intensive Medicine (ESICM) in 2016, (3) age ≥ 16 and ≤ 65 years old, and (4) agreement (by the subjects or their legal representatives) to enter the study and sign the informed consent form. Patients were excluded from the study if (1) they had previous organ failure, (2) they had previous immunological disorders, (3) they had a history of blood system diseases, or (4) they did not want to be included in the study. This study conformed to all the guidelines and principles stated in the Declaration of Helsinki.

### RNA-seq

The blood samples of each patient were sent for mRNA and miRNA sequencing at the same time. mRNA and miRNA sequencing were performed with the assistance of BGI (Shenzhen, China).

Briefly, total RNA was extracted from peripheral blood cells using TRIzol (Invitrogen, Carlsbad, CA, USA), and the RNA integrity number (RIN) was qualified and quantified with an Agilent 2100 bioanalyzer (Agilent, Santa Clara, USA). For quality control, mRNA had to meet the requirement of 28S/18S > 1, while miRNA had to meet the requirement of 28S/18S > 1.5.

After removal of ribosomal RNA (rRNA) from total RNA and solid-phase reversible immobilization (SPRI) bead purification, the RNA was fragmented into small pieces according to the kit manufacturer’s protocol. Afterwards, the fragmented RNA was reverse-transcribed into cDNA and amplified with polymerase chain reaction (PCR) to create a cDNA library. Quality control and quantification of the libraries were performed with an the Agilent 2100 bioanalyzer and real-time quantitative PCR (qPCR) (TaqMan Probe). The qualified libraries were subjected to mRNA sequencing on a DNBSEQ platform (BGI-Shenzhen, China).

After quality control and quantification, 1 μg of total RNA for each sample was prepared to construct a miRNA library. The total RNA was purified by electrophoretic separation, and small RNA regions that corresponded to the 18–30 nt bands in the marker lane (14–30 ssRNA ladder marker, TAKARA) were recovered. After annealing, the adapter-ligated small RNAs were transcribed into cDNA using SuperScript II Reverse Transcriptase (Invitrogen, Carlsbad, CA, USA), and the products were enriched via several rounds of PCR. The PCR products were screened by agarose gel electrophoresis for binding to target fragments of 110–130 bp and then purified with a QIAquick Gel Extraction Kit (Qiagen, Valencia, CA). The miRNA libraries were qualified using an Agilent 2100 Bioanalyzer and quantified via qPCR (TaqMan Probe). The final ligation PCR products were subjected to miRNA sequencing on a BGISEQ-500 platform (BGI-Shenzhen, China).

The sample reads were trimmed to remove reads with an unknown base (N) content greater than 5%, adapters and low-quality bases using Trimmomatic software and aligned with the reference genome using HISAT and Bowtie2 software.

### Differential gene and miRNA expression analysis

The data were divided into the sepsis group and the healthy control group. Bioinformatics analysis was performed according to the specific workflow of the online platform iDEP0.9^18^. The raw data were normalized with edgeR (V4.0)^19^, the expression values were converted by the log2(CPM + 4) values, genes with low-quality values were removed, and the preliminary DEGs and differentially expressed miRNAs (DEMs) were analysed using DESeq2^20^ iDEP0.9 with thresholds of a false discovery rate (FDR) < 0.05 and a log2 (fold change) (log2FC) value > 2. Afterwards, the top 20 miRNAs in ascending order of by FDR were selected as DEMs in sepsis. To further explore the potential miRNA regulators of core target genes, samples from the same patients were subjected to RNA-seq analysis to facilitate calculation of the relationships between miRNAs and target genes. For a regulatory relationship to be accepted, at least two conditions had to be met. First, complementary base pairing had to occur between the miRNA and mRNA. miRWalk3.0^21^ (http://mirwalk.umm.uniheidelberg.de/) was used to predict the potential target genes of DEMs in view of the principle of base pairing. Second, according to the mechanism of mRNA inhibition by miRNAs, there had to be a negative correlation between the expression values of the miRNA and the mRNA, the screening criteria were defined as a P value < 0.05 and a cor value < − 0.4. Among the preliminary DEGs, genes with a negative correlation with the DEMs were identified by OmicShare (https://www.omicshare.com/), and intersected with the predicted target genes of DEMs. The intersecting genes were defined as the final DEGs (hereafter referred to as DEGs).

### Enrichment analysis of DEG function

To further understand the integrated information on the DEGs from a broad perspective, the Metascape database (https://metascape.org/) was employed to conduct DEG Gene Ontology (GO) analysis, gene–disease association analysis^22^, and gene-organization distribution analysis^23^. The Metascape database integrates multiple authoritative data resources, such as the GO, Kyoto Encyclopedia of Genes and Genomes (KEGG), UniProt and DrugBank. Metascape provides provide comprehensive and detailed information about each gene and can be used to complete not only pathway enrichment and bioprocess annotation but also gene-related protein network analysis, gene–disease association analysis, and gene-organization distribution analysis.

### Construction of a miRNA–mRNA–PPI network

A PPI network was constructed based on previous research. In a PPI network, proteins with an interaction relationship are connected. If a specific target protein has more connections than other proteins, it is located at the core of the network. Therefore, researchers can infer whether a gene has potential research value on the basis of the network. The DEGs were subjected to PPI analysis using the STRING database (https://string-db.org/)^24^. In this study, the lowest value of the connection strength parameter between two proteins was 0.4. To ensure the reliable prediction, only experimentally verified results were included. The potential miRNA–mRNA relationships were predicted according to the posttranscriptional regulation mechanism and the negative correlation between miRNA and mRNA. On the basis of the above PPI network, the core genes were added to the miRNA–mRNA regulation relationships to provide potential clues for follow-up mechanistic research. The miRNA–mRNA–PPI network was visualized with OmicShare (https://www.omicshare.com/) to screen potential core genes.

### Hub gene survival analysis

The GSE65682 dataset^25^ was downloaded from the Gene Expression Omnibus (GEO) database (https://www.ncbi.nlm.nih.gov/geo/) and included clinical information such as gene expression data and survival time for 479 patients with sepsis in the ICU, including 365 sepsis survivors. The patients were divided into a high-expression group and a low-expression group according to the specific gene expression values. The survival data of patients with sepsis in GSE65682 were applied to conduct survival analysis for the core genes in the miRNA–mRNA–PPI network, and the survival curve was generated with GraphPad Prism (version 7.0) software, and the potential hub genes related to the prognosis of sepsis were selected. The log rank test was used for statistical analysis, and P < 0.05 was considered to indicate statistical significance.

### Negative regulation of miRNA–mRNA pairs

To understand the mechanism of the six hub genes in sepsis and the miRNAs involved in their regulation, directed network analysis between the core genes and the top 20 DEMs was performed using OmicShare tools (https://www.omicshare.com/tools). The regulatory relationships between six specific hub genes and upstream miRNAs were derived from the local network module described above (see above for relevant screening conditions).

### 10 × single-cell RNA sequencing and data analysis

Five PBMC were collected from 2 healthy controls, 1 systemic inflammatory response syndrome (SIRS) patient and 2 septic patients, and were subjected to single-cell RNA-seq analysis (10 × Genomics). The 10 × Genomics platform applied microfluidic technology according to the manufacturer’s protocol. The experimental data were further quality controlled for quality based on a preliminary quality control step with Cell Ranger to exclude data from mutliplets, doublets, or unbound cells. Cells with gene numbers and unique molecular identifier (UMI) numbers within the mean ± 2 standard deviations (SDs) and cells with fewer than 20% UMIs mapped to mitochondrial genes were considered high-quality cells. The results were visualized in two-dimensional space by t-distributed stochastic neighbour embedding (tSNE; nonlinear dimensionality reduction).

### Statistical analysis

The clinical data of the subjects were analysed with SPSS 22.0 software. Continuous variables are expressed as the mean ± SD and were analysed with unpaired Student’s *t* test; the chi-square test was adopted for categorical variables, and a *P* value < 0.05 was considered to indicate statistical significance.

### Ethics approval

This study was approved by the ethics committee of the Affiliated Hospital of Southwest Medical University (NO. ky2018029), and written informed consent was obtained from all participants all or their legal designates. The clinical trial registration number is ChiCTR1900021261. This study conformed to all the guidelines and principles stated in the Declaration of Helsinki.

### Consent for publication

No individual participant data are reported that would require consent from the participant to be published.

## Results

### Subjects’ clinical characteristics

**Figure 1:**
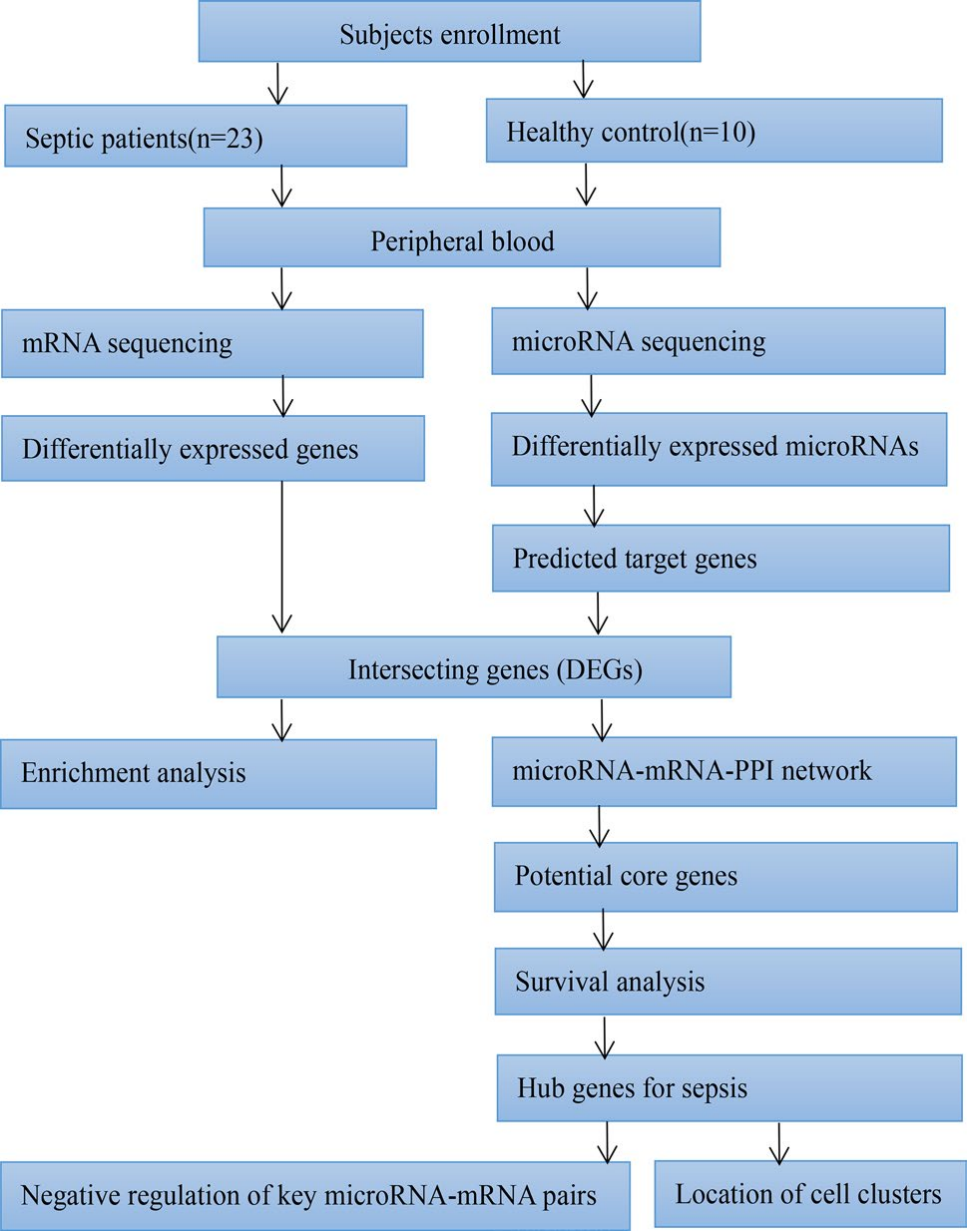
Experimental flow chart of this study. RNA-seq was applied to sequence mRNAs and miRNAs in the peripheral blood of a total of 33 subjects. Combined with bioinformatics, this analysis was used to screen out the key genes and corresponding miRNAs in sepsis.

The experimental flow chart of this study is shown in Fig. 1. A total of 23 patients with sepsis and 10 healthy volunteers were recruited for the current study. As the clinical information shown in Table 1, there were no significant differences in age, sex, or platelets (PLT) counts, alaninetransaminase (ALT), aspartate aminotransferase (AST), or creatinine (Crea) between the septic patients and the healthy controls, but considerable differences were found in white blood cell (WBC) counts, neutrophils counts, neutrophil/lymphocyte ratios (NLRs) and haemoglobin (HGB) levels. In addition, the levels of procalcitonin (PCT), prothrombin time/international normalization ratio (PT-INR), and lactic acid (LAC) were considerably higher than normal in the sepsis group. Blood cultures were positive in 12 (52%) sepsis cases, and gram-negative bacteria were the predominant microorganism (8, 66%). In addition, surgical sites were the major primary sites of infection (10, 43%). Compared with the healthy controls, the Charlson Comorbidity Index (CCI) that was used to predict 10-year survival in patients with multiple comorbidities, was higher and statistically significant in sepsis.

**Table 1:** Clinical information of the subjects.

Clinical variable	Healthy control (n = 10)	Sepsis (n = 23)	*P* value
Age (years)	50.7 ± 2.181	56.65 ± 3.621	0.3
Gender (F/M)	4/6	7/16	0.59
WBC (× 10^9^/L)	6.843 ± 0.628	13.8 ± 1.57	0.0076
NEU (× 10^9^/L)	4.141 ± 0.4236	12.19 ± 1.557	0.0021
PLT (× 10^9^/L)	215.3 ± 15.44	172.3 ± 20.13	0.19
NLR	2.04 ± 0.1536	23.83 ± 4.385	0.0028
HGB (g/L)	143.6 ± 8.755	104.1 ± 5.819	0.0007
ALT (U/L)	19.42 ± 1.912	86.22 ± 37.36	0.2517
AST (U/L)	20.55 ± 1.024	142.2 ± 56.79	0.1716
Crea (μmol/L)	67.15 ± 3.652	121.4 ± 26.79	0.1958
PT-INR	N/A	1.806 ± 0.429	
PCT (ng/mL)	N/A	29.65 ± 7.549	
LAC (mmol/L)	N/A	3.236 ± 0.69	
SOFA score	N/A	6.609 ± 0.8637	
**Primary sites of infection (n)**			
Surgical		10	
Dermatological infection		5	
Trauma		3	
Respiratory		4	
Urinary tract		1	
**Pathogens**			
Klebsiella pneumoniae		3	
Bacillus coli		3	
baumanii		1	
Streptococcus pneumoniae		3	
Acinetobacter Jones		1	
Pseudomyomyces white		1	
**Comorbidites (n)**			
Neoplasm		5	
Diabetes		3	
Hypertension		3	
CCI	0.5 ± 0.1667	2.87 ± 0.5528	0.0092

*SOFA score* Sequential Organ Failure Assessment score; *WBC* white blood count; *NEU* neutrophile; *PCT* procalcitonin; *PLT* platelet; *NLR* neutrophile/lymphocyte ratio; *HGB* hemoglobin; *LAC* lactic acid; *ALT* alaninetransaminase; *AST* aspartate aminotransferase; *Crea* creatinine; *PT-INR* prothrombin time/international normalization ratio; *CCI* Charlson Comorbidity Index.

### DEGs and DEMs in sepsis

To identify plasma prognostic biomarkers and potential mechanisms of sepsis, RNA-seq including mRNA and miRNA sequencing was utilized for each specimen simultaneously, mRNA sequencing for 33 subjects yielded the relative transcript levels of 20,391 genes. Furthermore, 1633 miRNAs were detected via miRNA sequencing.

**Figure 2:**
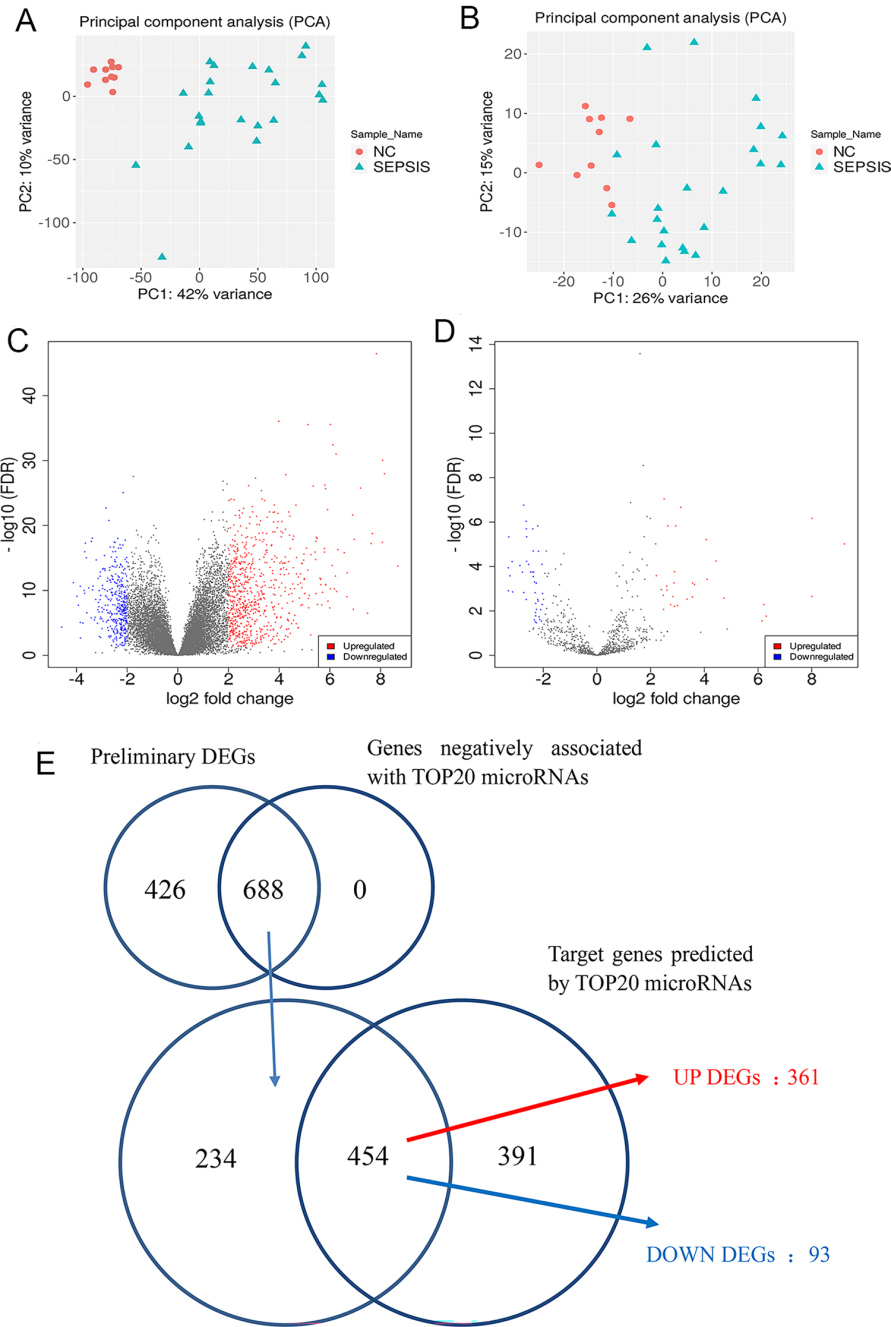
Bioinformatics analysis of RNA-seq data. (**A**) PCA of mRNAs. (**B**) PCA of microRNAs. Both panel A and panel B show that there was good consistency within the groups and a significant difference between the groups. (**C**) Volcano plot of mRNAs. (**D**) Volcano plot of miRNAs. (**E**) A workflow for screening DEGs. Red nodes, upregulated genes or miRNAs in sepsis; blue nodes, downregulated genes or miRNAs; gray nodes, genes or miRNAs with no considerable difference between the sepsis group and the NC group. Log2 fold change (log2FC) = 2, FDR < 0.05. *NC* the healthy control group, *SEPSIS* the sepsis group, *FDR* false discovery rate.

Afterwards, bioinformatics was used to analyse the above sequencing data to obtain the DEGs and DEMs. During normalizing, principal component analysis (PCA) was applied to remove the heterogeneous samples among the sepsis samples and control samples (Fig. 2A,B). A total of 1114 preliminary DEGs (767 upregulated) and 76 DEMs (45 downregulated) were initially screened out by DESeq2 (Fig. 2C,D) between the sepsis group and the healthy control group. According to the FDR values in ascending order, the top 10 upregulated and 10 downregulated miRNAs were considered as DEMs in sepsis (Table 2). Intersection analysis of the DEGs that were negatively associated with DEMs and the predicted target genes based on the DEMs with miRwalk3.0 yielded 454 differentially expressed genes (DEGs), the majority of which (361, 79.5%) were upregulated in sepsis. A detailed workflow for screening DEGs is shown in Fig. 2E.

**Table 2:** The top 20 DEMs between patients with sepsis and healthy controls.

DEMs	Log_2_FC	FDR	Regulation
hsa-miR-9-5P	9.218126562	9.61E−06	Up
hsa-miR-149-5P	4.431721624	5.72E−05	Up
hsa-miR-218-5P	4.103297275	0.000375427	Up
hsa-let-7c-5P	3.56709988	0.000556136	Up
hsa-miR-212-5P	3.63722981	0.000644706	Up
hsa-miR-153-3P	8.003693486	0.002226582	Up
hsa-miR-187-3P	4.736627079	0.002656874	Up
hsa-miR-129-5P	6.215312507	0.005233831	Up
hsa-miR-124-3P	6.307683489	0.017232704	Up
hsa-miR-219a	6.147353433	0.02806464	Up
hsa-miR-454-3P	−2.627679377	2.02E−06	Down
hsa-let-7f-5P	−2.613757362	4.60E−06	Down
hsa-miR-190b-5P	−2.175214826	2.05E−05	Down
hsa-miR-20a-5P	−2.620742801	5.87E−05	Down
hsa-miR-144-5P	−2.866873271	9.02E−05	Down
hsa-miR-196b-5P	−2.471814233	0.000177083	Down
hsa-miR-144-3P	−3.236825564	0.000263461	Down
hsa-miR-32-5P	−2.327794713	0.000494179	Down
hsa-miR-126-5P	−2.262718884	0.000571466	Down
hsa-miR-101-3P	−2.025473487	0.001640951	Down

*DEMs* differentially expressed miRNAs, *FC* fold change, *FDR* false discovery rate, *miRNAs* microRNAs.

### Enrichment analysis of DEG function

**Figure 3:**
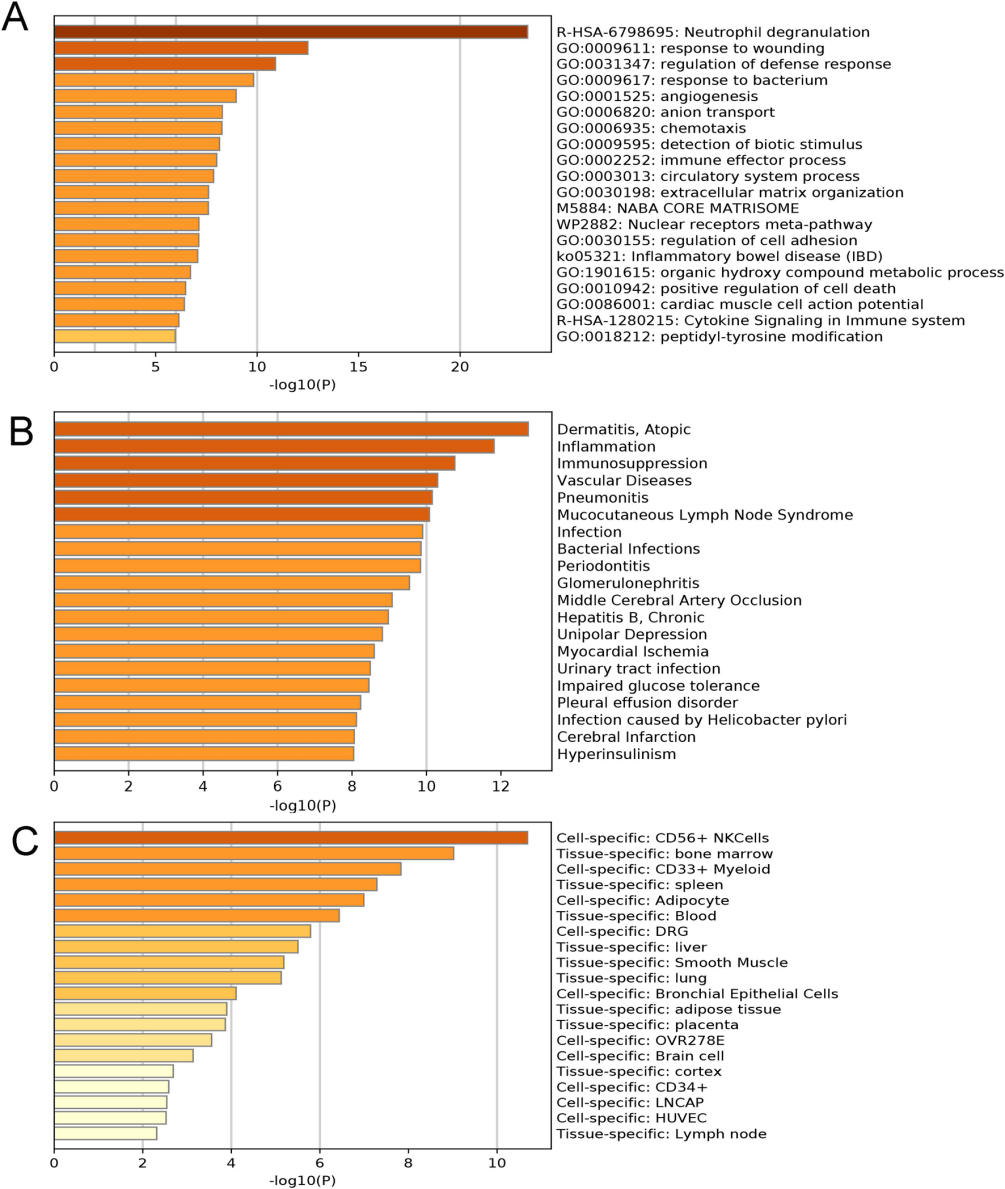
Enrichment analysis of DEGs via the Metascape database. (**A**) Biological processes associated with the DEGs between septic patients and healthy controls. (**B**) Diseases that may be associated with the DEGs. (**C**) Cell- or tissue-specific distribution of the DEGs. *DEGs* differentially expressed genes.

We deem it essential to comprehensively elucidate the biological functions of the DEGs. Enrichment analysis showed that the identified DEGs were significantly related to neutrophil degranulation, regulation of defence, response to bacteria, and chemotaxis (Fig. 3A). Gene–disease association analysis revealed that these genes may be involved in inflammation, immunosuppression, pneumonia, and bacterial infection (Fig. 3B). Furthermore, gene-organization distribution analysis revealed that these genes are mainly distributed in natural killer (NK) cells, the bone marrow, the spleen, adipocytes, the blood, and the liver (Fig. 3C).

### miRNA–mRNA–PPI regulatory network

**Figure 4:**
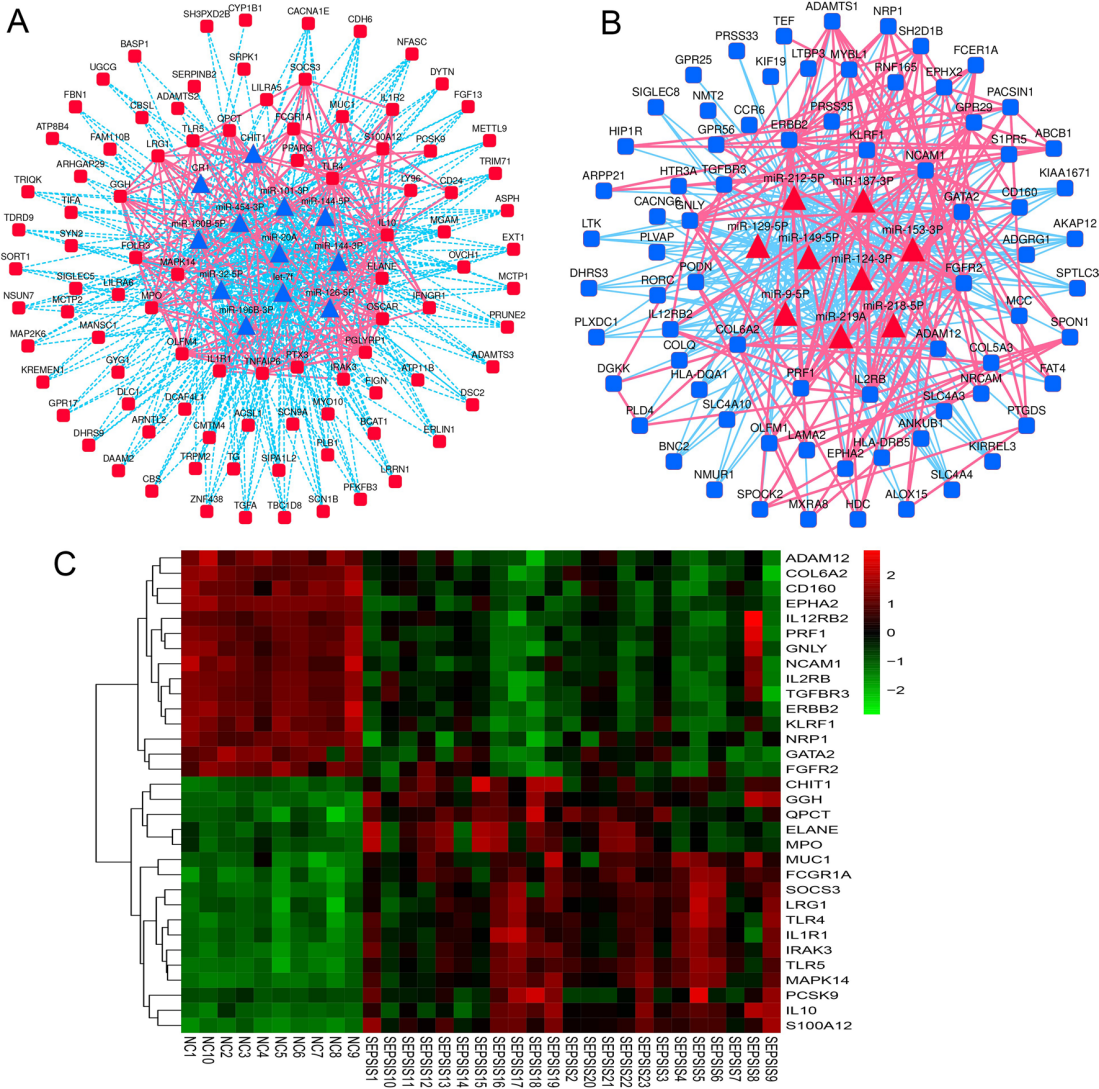
miRNA–mRNA–PPI regulatory network for identification of potential key genes. (**A**) miRNA–mRNA–PPI network of upregulated DEGs and downregulated DEMs. Red squares, upregulated DEGs; red lines, protein–protein interactions; blue triangles, downregulated DEMs; blue lines, miRNA–mRNA interactions. (**B**) miRNA–mRNA–PPI network of downregulated DEGs and upregulated DEMs. Blue squares, upregulated DEGs; red lines, protein–protein interactions; red triangles, downregulated DEMs; blue lines, miRNA–mRNA interactions. (**C**) Heatmap of the 30 potential key genes based on genes located at the centre of the miRNA–mRNA–PPI network. Data from controls are shown in blue and data from septic patients are shown in red. *PPI* protein–protein interaction, *DEMs* differentially expressed miRNAs, *DEGs* differentially expressed genes, *NC* healthy control group.

The DEMs and DEGs were submitted to the STRING database (https://string-db.org/), and the OmicShare (https://www.omicshare.com/). A miRNA–mRNA–PPI regulatory network for sepsis was constructed (Fig. 4A,B), and the 30 potential key genes located at the centre of the miRNA–mRNA–PPI network were screened out as the potential core genes involved in sepsis and presented in a heatmap (Fig. 4C). In the two groups of different expression trend modules, the core genes such as CD160, GATA2, GNLY, IL2RB and TGFBR3 were downregulated in the sepsis group, while genes such as ELANE, IL1R1, TLR5, FCGR1A, MAPK14 and PCSK9 were upregulated.

### Hub gene survival analysis

**Figure 5:**
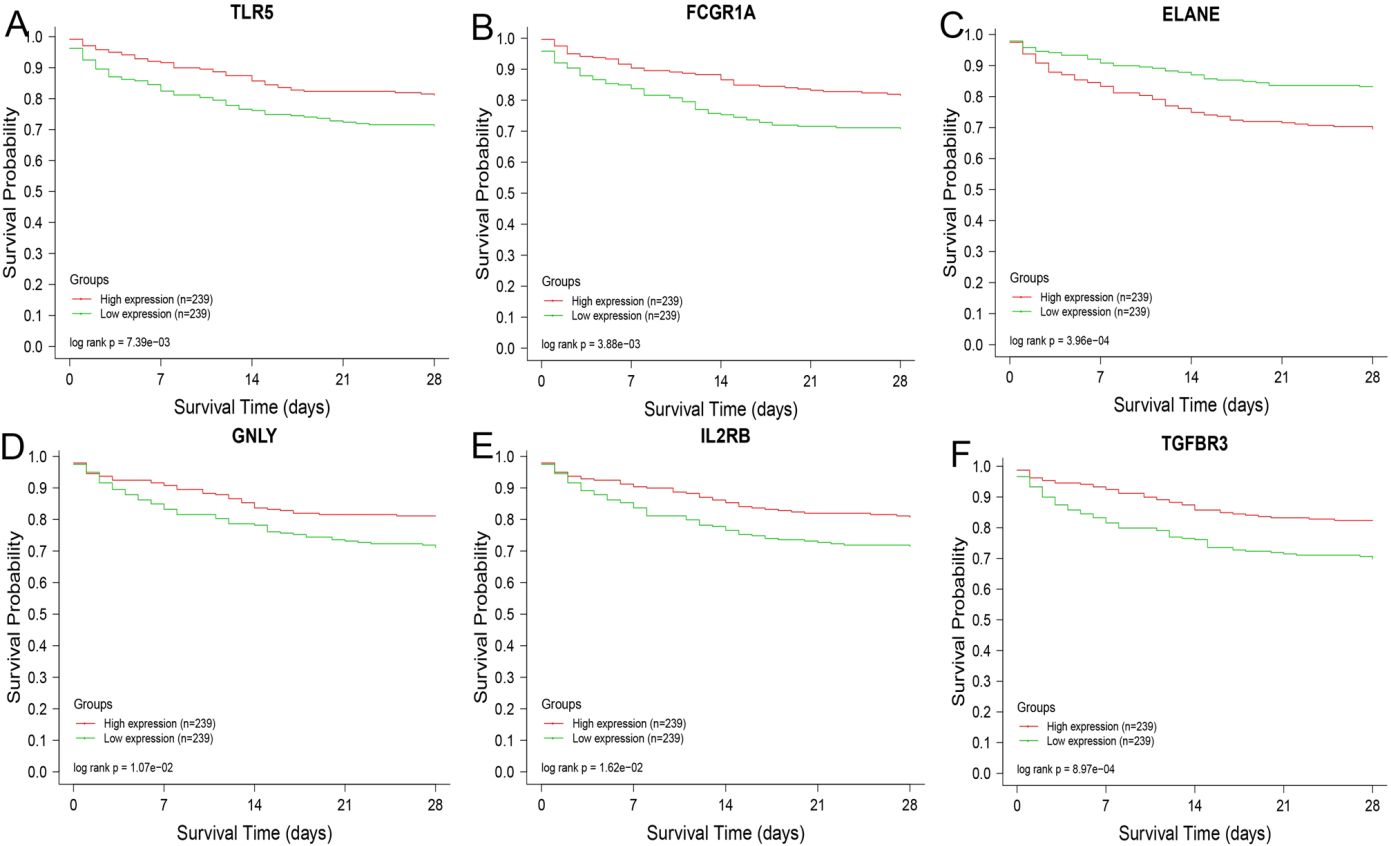
Identification of core genes for sepsis. The survival analysis of the potential core genes was based on dataset GSE65682. The relationships between the expression levels of hub genes and the 28-day survival times of the septic patients were explored. Higher expression of the genes TLR5 (**A**), FCGR1A (**B**), GNLY (**D**), IL2RB (**E**) and TGFBR3 (**F**) was associated with higher survival rates of patients with sepsis (*P* < 0.05). Higher expression of the gene ELANE (**C**) was associated with a poorer outcome in septic patients (*P* < 0.05).

Based on the data from GSE65682, we explored associations between the potential core genes and sepsis outcomes, Survival analysis showed that the genes TLR5, FCGR1A and ELANE whose expression was upregulated in sepsis, and the genes GNLY, IL2RB and TGFBR3, whose expression was downregulated in sepsis, were significantly associated with sepsis outcomes, Higher expression of the genes TLR5, FCGR1A, GNLY, IL2RB and TGFBR3 was associated with the better the clinical outcomes in patients with sepsis (*P* < 0.05). The relationship between the gene ELANE and sepsis outcomes showed the opposite trend (*P* < 0.05) (Fig. 5A–F). Consequently, the genes TLR5, FCGR1A, ELANE, GNLY, IL2RB and TGFBR3 genes were ultimately identified as hub genes in sepsis.

### Negative regulation of hub miRNA–mRNA pairs

**Figure 6:**
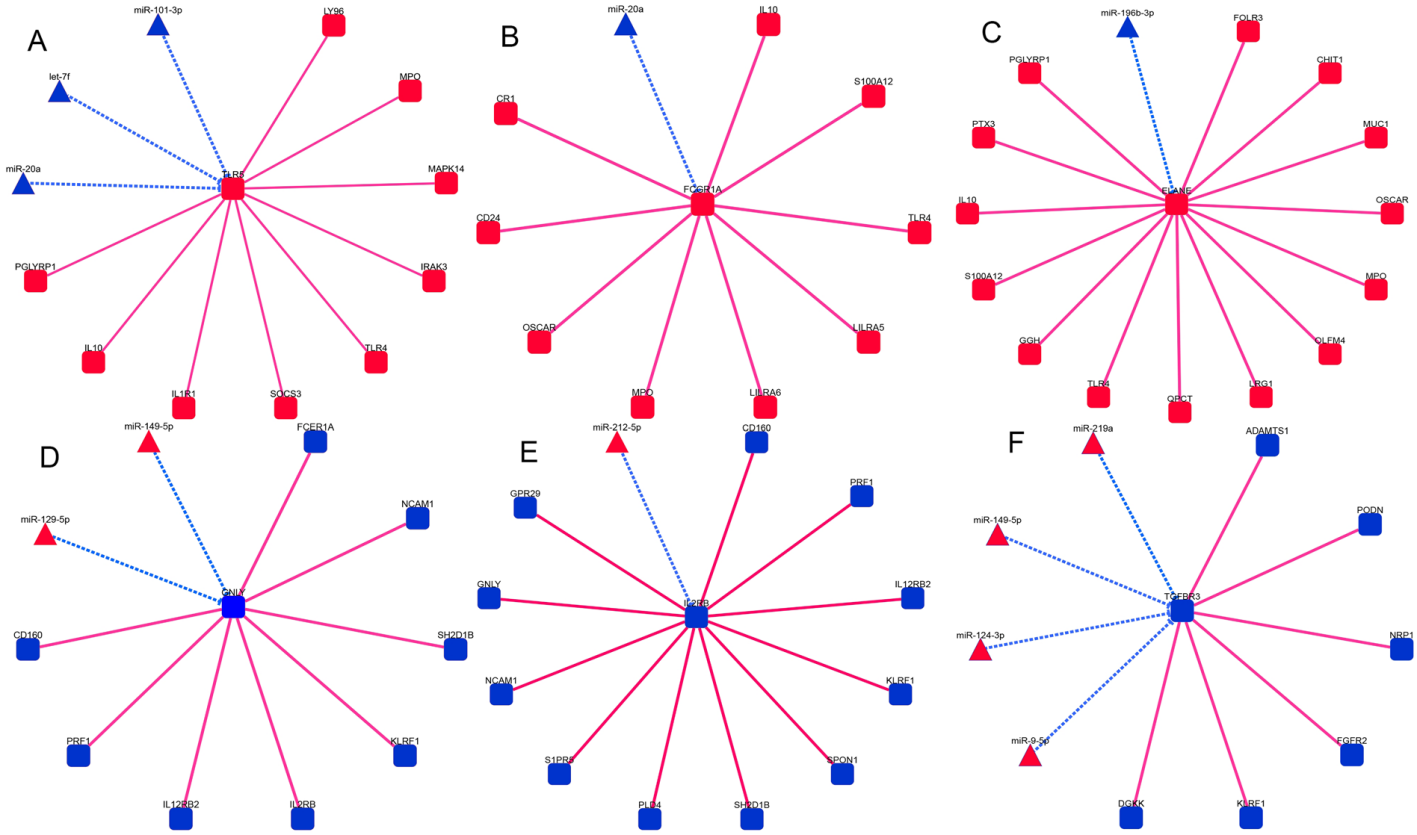
Graph of the network between the hub genes and miRNAs. (**A**) TLR5 may be negatively regulated by miR-20a, miR-101-3p, and let-7f. (**B**) miR-20a may negatively regulate FCGR1A. (**C**) Negative regulatory relationship between ELANE and miR-196b-3p. (**D**) GNLY may be negatively regulated via miR-129-5p, miR-149-5p. (**E**) miR-212-5p may negatively regulate IL2RB. (**F**) TGFBR3 may be negatively regulated by miR-219a, miR-149-5p, miR-124-3p, miR-9-5p.

Directed network analysis with OmicShare (https://www.omicshare.com/) was used to analyse the regulatory relationships between hub genes and miRNAs, and a directed network diagram was drawn. The miRNAs associated with these core biomarkers were identified and are presented in Fig. 6A–F.

### Location of hub genes in cell clusters

**Figure 7:**
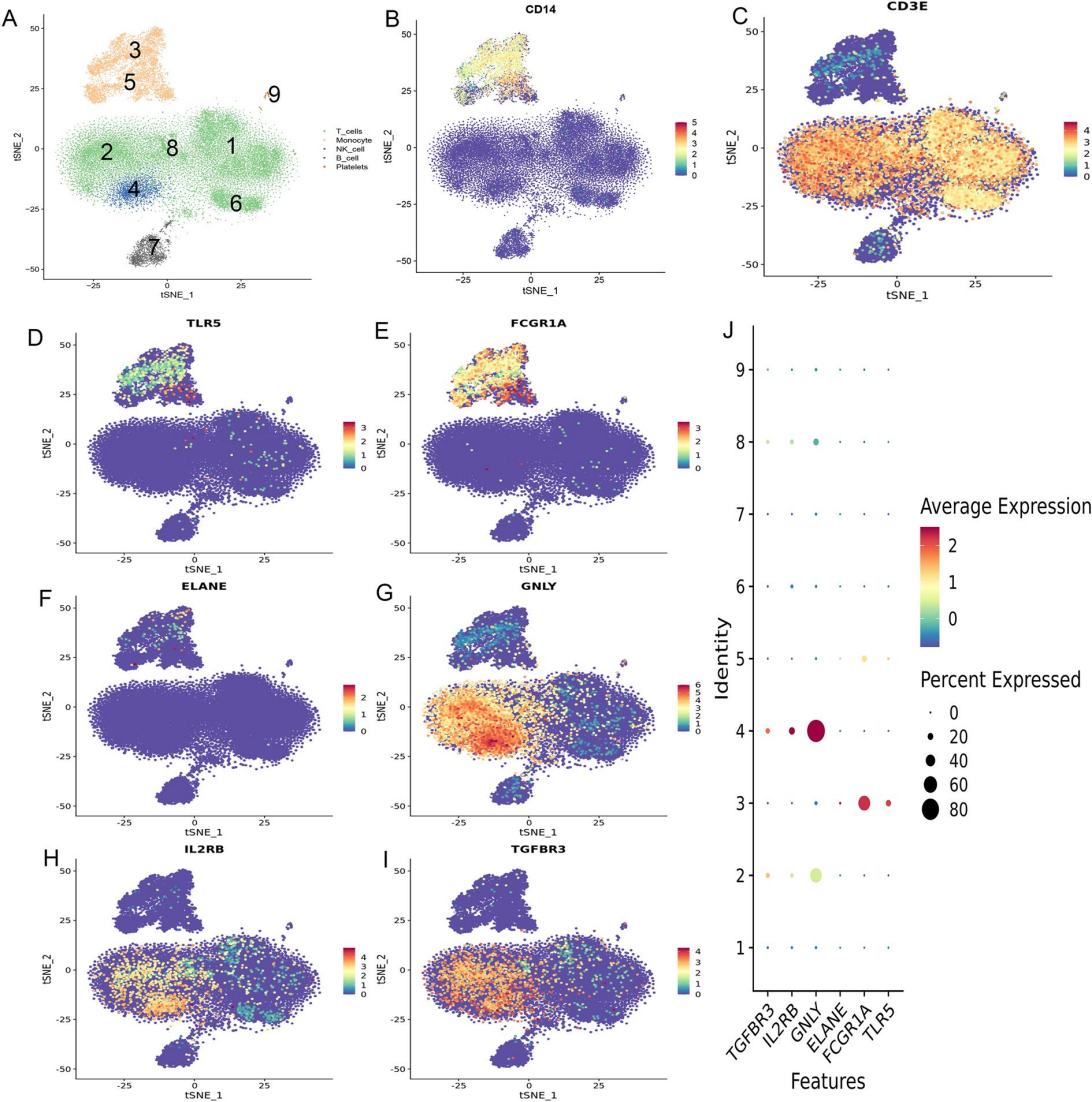
Locations of hub genes in cell clusters. (**A**) Nine transcriptionally distinct cell clusters were distinguished. (**B**) CD14 was the hallmark of monocytes. (**C**) CD3E was a characteristic marker of the NK cells and T cells. (**D**–**F**) The genes TLR5, FCGR1A and ELANE were distributed in monocytes. (**G**–**I**) The genes GNLY, IL2RB and TGFBR3 were expressed in NK cells and T cells. (**J**) Bubble plots showing the expression abundance values and ratios of each hub gene in different cell clusters.

We compared gene expression patterns in PBMCs among healthy controls, SIRS patients and septic patients and identified 9 transcriptionally distinct cell clusters (Fig. 7A), The markers CD14and CD3E represented monocytes and NK-T cells, respectively (Fig. 7B,C). The genes TLR5, FCGR1A and ELANE genes were mainly expressed in macrophages (Fig. 7D–F), while the genes GNLY, IL2RB and TGFBR3 genes were expressed specifically in T cells and NK cells (Fig. 7G–I). The expression abundance values and ratios of each hub gene in different cell clusters are shown in Fig. 7J. These findings lay a foundation for subsequent mechanistic studies.

## Discussion

RNA-seq, also referred to as transcriptome sequencing, is a newly developed technique for transcriptome analysis that use deep sequencing technology and can quantitatively detect RNA expression levels^12^. RNA-seq can be applied to identify DEGs in healthy and diseased tissues and provide a platform for further study of the mechanism of sepsis. Sepsis is a health- and life-threatening condition. Despite early administration of antibiotics and the improvements in organ support, the rates of mortality remain high among patients with sepsis. The development of immune response and the prognosis of sepsis are the focuses of medical research. The aim of the present study was to distinguish molecular differences between patients with sepsis and healthy controls and to determine associations with sepsis outcomes. We conducted RNA-seq (including mRNA and miRNA sequencing) on 23 patients with sepsis and 10 healthy controls and distinguished 454 DEGs (361 upregulated) and 20 DEMs in patients with sepsis compared to healthy volunteers. Based on clinical phenomena, hundreds of DEGs were screened, and functional enrichment analysis was carried out to understand the overall change characteristics of the genes. To further explore which genes play keys role in sepsis, a miRNA–mRNA–PPI regulatory network was constructed through integrated transcriptomics analysis for the above DEGs and 20 DEMs. We obtained dozens of potential core targets and determined their mutual regulatory relationships via network analysis. Moreover, survival curves were analysed for the potential core targets, and six hub genes were ultimately identified, including the upregulated genes TLR5, FCGR1A and ELANE and the downregulated genes GNLY, IL2RB and TGFBR3. Moreover, these genes are highly related to the prognosis of sepsis. In addition, the biological functions of the core genes were probably associated with bacterial infection, inflammation, immunosuppression and chemotaxis. The localization of these hub genes in PBMCs was further clarified by single-cell sequencing.

miRNAs have been proposed as to be good biomarkers because they are stably present in biofluids and biospecimens, including blood, urine, and saliva; this availability enables relatively easy collection and analysis using different methods, such as RNA-seq and qPCR^26,27^. Abnormal miRNA expression is correlated with the severity of sepsis and may serve as a potential diagnostic and prognostic biomarker in sepsis^16,28^. A variety of miRNAs, including miR-150, miR-133a, miR-223 and miR-23a have been described to play roles in sepsis or sepsis-related organ injury^29–31^. Furthermore, based on integrated analyses of miRNAs and mRNA, miR-106b-5p, miR-128-3p, and miR-144-3p and their mRNA targets are new potential diagnostic and therapeutic indicators^13^. In the present study, we assessed the hub genes that bind with DEMs and found that they were negatively regulated by different miRNAs, providing beneficial opportunities for studying the pathogenesis of sepsis. We observed that the downregulated miRNAs miR-20a, miR-101-3p, let-7f and miR-196B-3p and the upregulated miRNAs miR-212a, miR-129-5p, miR-149-5p, miR-219a, miR-124-3p and miR-9-5p potentially regulate these hub genes.

TLR5, a core member of the Toll-like receptors (TLRs) family, is a receptor of bacterial motile components that targets extracellular flagellin and thus induces inflammatory cytokines production and the immune response^32^. Previous studies have demonstrated that TLR5 not only participates in *H. pylori* infection^33^ and SIRS^34^, but also prevents systemic inflammation and liver damage caused by *B. pseudomallei* infection. TLR5 deficiency facilitates bacterial growth and dissemination^35^. Another paper has indicated that increased TLR5 expression on monocytes is associated with mortality in patients with sepsis^36^, which is not consistent with our founding that elevated TLR5 expression in peripheral blood is associated with attenuated mortality of septic patients. This discordance might be explained by the different samples used; more experiments are needed to confirm this hypothesis. FCGR1A, also named CD64, is expressed on most myeloid cells and is a high-affinity Fc receptor (FcγRI), that binds to monomeric IgG^37^. It participates in a number of functions, including phagocytosis, antigen presentation, and cytokine production^38^. Neutrophils FCGR1A is involved in tuberculosis (TB), regardless of HIV infection^39,40^, and can be used to differentiate TB from latent TB infection^41^. A previous study has indicated that neutrophil CD64 expression is an important diagnostic marker of infection and sepsis in hospital patients^42^. In the current study, elevated expression of FCGR1A in peripheral blood was associated with a good outcome in sepsis, and FCGR1A was mainly expressed in macrophages. The gene ELANE encodes an elastase that exists in neutrophils, and plays an important role in pathogen killing. A previous study found that ELANE was overexpressed in septic patients via gene expression profile analysis^43^, which is in accordance with our conclusions. Moreover, inhibition of ELANE-mediated histone H3 proteolysis contributes to mononuclear macrophage differentiation^44^. GNLY is a cytotoxic granular protein secreted by cytotoxic T lymphocytes and NK cells^45^ that exerts toxic effect on bacteria, fungi, parasites, and tumors^46^, GNLY acts as an immune alarmin and promotes antigen-presenting cell activation through TLR4^47^. GNLY is associated with the efficacy of pegylated-interferon-alpha therapy in Chinese patients with HBeAg-positive chronic hepatitis^48^, rheumatoid arthritis^49^, and *Mycoplasma pneumoniae* pneumonia^50^. IL2RB, a subunit of IL-2R, mediates signal transduction for IL-2R and IL-15R^51^, Mutations in human IL2RB result in immune dysregulation, cytomegalovirus (CMV) susceptibility^52^, reduced T reg frequency, and an abnormal NK compartment^53^. Gene expression profiling and bioinformatics analysis have indicated that IL2RB is weakly expressed in sepsis, which indicates that IL2RB may be a potential diagnostic tool for sepsis^54,55^. Our findings are consistent with this possibility. TGFBR3, also known as betaglycan, is a coreceptor of the TGF-β superfamily, and plays important roles in cardiomyocyte apoptosis^56^, renal cell carcinoma^57^, keratinocyte proliferation^58^, cervical carcinoma^59^, and angiogenesis^60^. To our knowledge, there is little evidence of a role of TGFBR3 in sepsis.

In summary, we comprehensively analyzed miRNA–seq, mRNA–seq and single-cell sequencing profiling and established an integrated miRNA–mRNA–PPI network to screen hub genes in sepsis, the potential hub genes TLR5, FCGR1A, ELANE, GNLY, IL2RB and TGFBR3 and miRNAs that are possible posttranscriptional and regulatory factors related to sepsis prognosis were screened out, the findings provide new prospects for exploration of the physiopathologic mechanisms, diagnosis, and treatment of sepsis. Nevertheless, some limitations of this study should be mentioned. First, this study was performed in a single centre with a small sample size; and we will increase the sample size in further research. Second, the mechanisms of the hub genes in sepsis must be validated in subsequent experiments, as they were not confirmed in the current study.

## Data Availability

The RNA-seq dataset analysed during the current study is available in the China National GeneBank DataBase (CNGBdb) and can be found below: https://db.cng.org/, under the accession: CNP0002611.
