# Author Correction: Identification of hub genes for adult patients with sepsis via RNA sequencing

**DOI:** 10.1038/s41598-022-10297-7

**Published:** 2022-04-13

**Authors:** Qian Zhang, Yingchun Hu, Peiyao Wei, Liu Shi, Lei Shi, Jianzhou Li, Yalei Zhao, Yunru Chen, Xi Zhang, Feng Ye, Xiaojing Liu, Shumei Lin

**Affiliations:** 1 Department of Infectious Diseases, The First Affiliated Hospital of Xi’an Jiaotong University, Xi’an, Shaanxi China; 2 Department of Infectious Diseases, The Affiliated Hospital of Southwest Medical University, Luzhou, Sichuan China; 3 Department of Emergency Medicine, The Affiliated Hospital of Southwest Medical University, Luzhou, Sichuan China

Correction to: *Scientific Reports* 10.1038/s41598-022-09175-z, published online 24 March 2022

The original version of this Article contained errors in Table 1 and its legend. The “clinical variables” rows, such as ALT (U/L), AST (U/L), Crea (μmol/L), PT-INR, PCT (ng/ml), LAC (mmol/L), CCI and their values were omitted from the Table.

Additionally, the value for Neoplasm was incorrect,

“4”

now reads:

“5”

In the legend of Table 1,

“*SOFA score* Sequential Organ Failure Assessment score, *WBC* white blood count, *NEU* neutrophile, *PCT* procalcitonin, *PLT* platelet, *NLR* neutrophile/lymphocyte ratio, *HGB* hemoglobin.”

now reads:

“SOFA score, Sequential Organ Failure Assessment score; WBC, White blood count; NEU, neutrophile; PCT, procalcitonin; PLT, platelet; NLR, neutrophile/lymphocyte ratio; HGB, hemoglobin; LAC, lactic acid; ALT, alaninetransaminase; AST, aspartate aminotransferase; Crea, creatinine; PT-INR, prothrombin time/international normalization ratio; CCI, Charlson Comorbidity Index.”

The original Table 1 and accompanying legend appear below.

**Table 1:** Clinical information of the subjects.

Clinical variable	Healthy control (n = 10)	Sepsis (n = 23)	*P* value
Age (years)	50.7 ± 2.181	56.65 ± 3.621	0.3
Gender (F/M)	4/6	7/16	0.59
WBC (×10^9^/L)	6.843 ± 0.628	13.8 ± 1.57	0.0076
NEU (×10^9^/L)	4.141 ± 0.4236	12.19 ± 1.557	0.0021
PLT (×10^9^/L)	215.3 ± 15.44	172.3 ± 20.13	0.19
NLR	2.04 ± 0.1536	23.83 ± 4.385	0.0028
HGB (g/L)	143.6 ± 8.755	104.1 ± 5.819	0.0007
ALT (U/L)	19.42 ± 1.912	86.22 ± 37.36	0.2517
AST (U/L)	20.55 ± 1.024	142.2 ± 56.79	0.1716
Crea (μmol/L)	67.15 ± 3.652	121.4 ± 26.79	0.1958
PT-INR	N/A	1.806 ± 0.429	
PCT (ng/ml)	N/A	29.65 ± 7.549	
LAC (mmol/L)	N/A	3.236 ± 0.69	
SOFA score	N/A	6.609 ± 0.8637	
**Primary sites of infection (n)**			
Surgical		10	
Dermatological infection		5	
Trauma		3	
Respiratory		4	
Urinary tract		1	
**Pathogens**			
Klebsiella pneumoniae		3	
Bacillus coli		3	
Baumanii		1	
Streptococcus pneumoniae		3	
Acinetobacter Jones		1	
Pseudomyomyces white		1	
**Comorbidites (n)**			
Neoplasm		5	
Diabetes		3	
Hypertension		3	
CCI	0.5 ± 0.1667	2.87 ± 0.5528	0.0092

*SOFA score* Sequential Organ Failure Assessment score, *WBC* white blood count, *NEU* neutrophile, *PCT* procalcitonin, *PLT* platelet, *NLR* neutrophile/lymphocyte ratio, *HGB* hemoglobin.

The original Article has been corrected.

